# Mediastinal extension of a complicated pancreatic pseudocyst; a case report and literature review

**DOI:** 10.1186/1752-1947-1-12

**Published:** 2007-04-25

**Authors:** Umar Sadat, Asif Jah, Emmanuel Huguet

**Affiliations:** 1 Department of Surgery, Addenbrooke's Hospital, Cambridge University Hospitals NHS Foundation trust, Cambridge, UK

## Abstract

**Background:**

Mediastinal pancreatic pseudocyst is a rare complication of acute or chronic pancreatitis.

**Case presentation:**

This case report describes the management of a difficult case of pancreatic pseudocyst with a mediastinal extension in a patient having chronic pancreatitis. Different management strategies were used until complete resolution of this complex pseudocyst occurred using open surgical cystogastrostomy.

**Conclusion:**

Despite the availablity of different minimally invasive techniques to treat pancreatic pseudocysts, management of complex mediastinal pseudocyst may still require open surgical drainage procedures.

## Background

Mediastinal pancreatic pseudocyst is a rare complication of acute or chronic pancreatitis. Since its first description in 1951, approximately 50 cases have been reported in the world literature. We report a case of mediastinal pseudocyst associated with alcoholic pancreatitis successfully treated with cystogastrostomy.

## Case presentation

**Figure 1:**
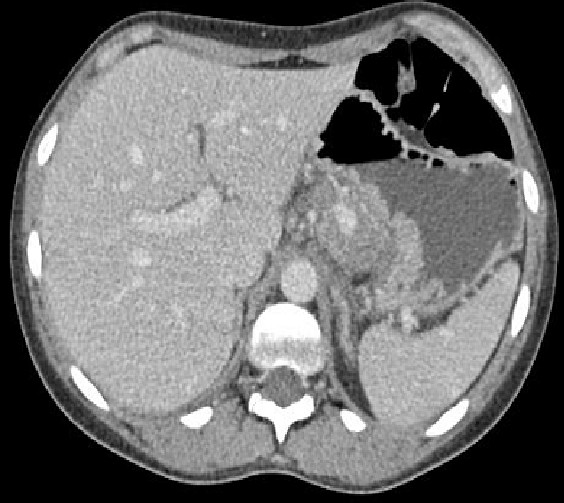
Contrast enhanced CT-scan showing left gastric artery pseudoaneurysm.

A 22-year-old female patient with a known history of chronic alcohol abuse was referred to hepato-biliary unit with recurrent upper abdominal related to chronic pancreatitis. Prior to this she has had multiple hospital admissions in previous one year with similar symptoms. In the past, ultrasonography had excluded gallstones and a CT scan 12 months ago had shown normal-looking pancreas. Her most notable symptomatic episode was 4 months ago when she was admitted with left lateral chest pain and dyspnoea. A CT-scan although excluded a pulmonary embolism but incidentally revealed a left gastric artery pseudoaneurysm (Figure 1) along with pancreatic calcifications. Attempt to embolise the aneurysm was unsuccessful, because of difficult anatomy, following which she underwent a laparotomy and ligation of the pseudoaneurysm.

**Figure 2:**
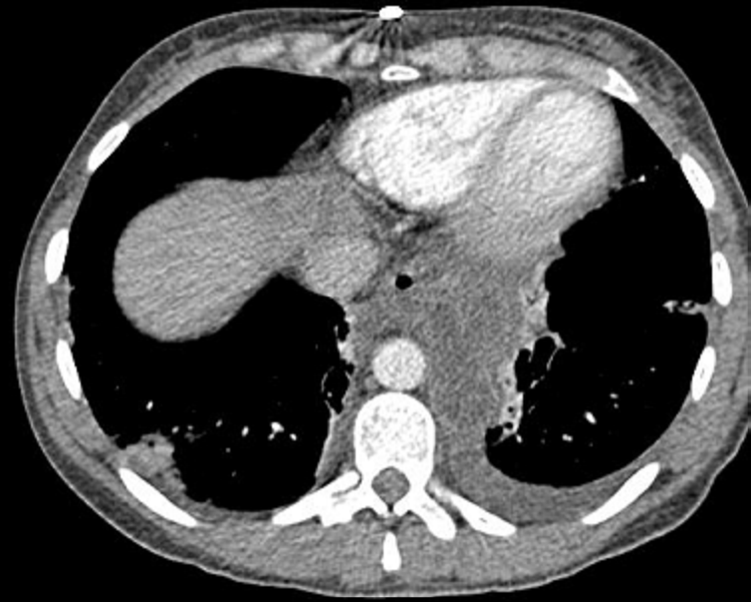
CT-scan showing pancreatic pseudocyst and its mediastinal extension.

When she presented to us she was in distress with epigastric pain radiating to the back and prominent dysphagia for solids. Her blood picture showed Leucocytosis and slightly elevated serum amylase and Gamma-GT. CT scan showed large fluid collection in the lesser sac tracking behind the oesophagus and into lower mediastinum extending up to the level of carina (Figure 2). The fluid collection had an enhancing wall and was associated with previously detected features of calcific chronic pancreatitis. The bulk of the cyst was in the mediastinum and was associated with left sided pleural effusion. An Endoscopic Ultrasound (EUS) confirmed the findings and cyst was aspirated to dryness. Cyst fluid biochemistry showed high amylase levels (18088 u/l) thus confirming the lesion to be a pancreatic pseudocyst. After initial resolutions of symptoms following cyst aspiration she became symptomatic again within a few days and therefore a surgical cystogastrostomy was performed. Post-operative recovery was uneventful and a follow-up CT scan 3 months after surgery showed completed resolution of the cyst.

## Conclusion

Mediastinal Pseudocysts are caused by rupture of the pancreatic duct posteriorly into the retroperitoneal space and subsequent tracking of the fluid in to mediastinum. In the majority of patients, the pancreatic fluid enters the mediastinum through the esophageal or aortic hiatus [1] leading to pseudocyst formation in the posterior mediastinum. A fistulous track often connects the larger thoracic component to the abdominal part of the cyst. Other less frequent sites of entry into the mediastinum are the foramen of Morgagni, the inferior vena cava hiatus and direct penetration of the diaphragm [1,2]. Mediastinal pseudocysts may rupture into the pleural space producing pleural effusion or may extend further into the neck.

The diagnosis of pancreatic pseudocysts should be suspected in the setting of pancreatitis. They are more likely to develop and liable to persist in patients with chronic pancreatitis as in our case. Mediastinal pseudocysts may have no specific symptoms or may be associated with back pain, dysphagia or oesophageal reflux. Our patient had marked gastro-oesophageal reflux symptoms that have been previously described with mediastinal pseudocysts. It is probably due to ineffectiveness of anti-reflux mechanisms of the diaphragmatic crura, widening of hiatus, and loss of gastro-oesophageal angle. As with our case, pleural effusion is present in majority of cases. It has been postulated that the pleural effusion is due to lymphatic obstruction due to peri-cystic inflammation.

Frequently, diagnosis of mediastinal pseudocyst is made on cross-sectional imaging. CT scan is the investigation of choice which demonstrates the presence of thick walled, cystic lesion in the posterior mediastinum. Additionally, MRI scan has been shown to be useful in demonstrating the fistulous tract extending to pancreas [3]. Fluid from a mediastinal cyst under ultrasound guidance showing high amylase level can confirm the diagnosis of a mediastinal pseudocyst [4].

The ideal management of mediastinal pseudocysts is controversial and depends upon the exact location, underlying aetiology, ductal anatomy, size of the pseudocyst and expertise available. Pancreatic pseudocysts, irrespective of the location are initially treated conservatively. However, spontaneous regression of mediastinal cysts is rare [5].

Several treatment approaches have been described. Medical management has been anecdotally reported to be successful and is aimed at minimizing the pancreatic exocrine secretions. Somatostatin or its analogues have been used but this usually requires prolonged therapy [6]. Total parenteral nutrition has also been reported to result in resolution of the cyst [7]. Successful resolution of mediastinal pseudocysts with 5-months of therapy with the mucolytic agent bromhexine hydrochloride has also been reported [8].

Endoscopic interventions with their obvious advantages for the patient are increasingly used as the first modality of treatment. Endoscopic procedures are better tolerated by the patient and have the advantage of not precluding any future surgery, if required. Trans-gastric drainage and/or deployment of stent under EUS-guidance has been shown to highly successful [9]. However, the pseudocyst has to be bulging in to the viscus for this to be possible. In our case, EUS-guided stent deployment was considered but was deemed inappropriate because cyst was not in close relationship with stomach. Trans-oesophageal stent deployment although has been reported in the literature was not considered in this case because of obvious theoretical risk of oesophageal perforation and/or stricture. There have also been reports of resolution of pseudocysts following transpapillary pancreatic stenting [2,10]. ERCP especially has played a revolutionary role in managing the complications of chronic pancreatitis and in defining the ductal anatomy in up to 80% of all pancreatic pseudocysts. [11]

The surgical procedures described for pseudocysts are varied and range from or external or internal drainage to pancreatic resections. Surgery should be considered in symptomatic patients and if there are associated complications such as infection, obstruction, rupture, or hemorrhage. Approaches include cystogastrostomy or cystojejunostomy carry a low recurrence rate. Increasingly, surgical drainage procedures are been performed laparoscopically with low morbidity and faster recovery. In our case, laparoscopic approach was not used due to previous upper abdominal surgery.

## Conflict of interests

The author(s) declare that they have no competing interests.

## Authors' contributions

All the authors have been involved in literature search, writing and final reviewing of this manuscript.
